# Chemical medium-range order in a medium-entropy alloy

**DOI:** 10.1038/s41467-022-28687-w

**Published:** 2022-02-23

**Authors:** Jing Wang, Ping Jiang, Fuping Yuan, Xiaolei Wu

**Affiliations:** 1 State Key Laboratory of Nonlinear Mechanics, Institute of Mechanics, Chinese Academy of Sciences, Beijing, 100190 China; 2 School of Engineering Science, University of Chinese Academy of Sciences, Beijing, 100049 China

**Keywords:** Metals and alloys, Mechanical properties

## Abstract

High-/medium-entropy alloys (H/MEA) have the inherent local chemical order. Yet, as a structural link between the incipient short-range order and mature long-range counterpart, the chemical medium-range order (CMRO) is conjectural and remains open questions as to if, and what kind of, CMRO would be produced and if CMRO is mechanically stable during plastic deformation. Here, we show compelling evidences for CMRO in an Al_9.5_CrCoNi MEA. Specifically, the electron diffraction under both [$112$] and [$013$] zone axis show the definite spots for CMRO of lattice periodicity. CMRO entities are seen directly of medium-ranged in sizes by using dark-field imaging, along with the tendency towards like-pair avoidance and unlike-pair preference based on atomic-resolution EDS mapping. These findings substantiate CMRO with a realistic structural picture in view of crystal periodicity and chemical species occupation, shedding light on understanding the microstructural link at an extended length scale beyond the short-range order.

## Abstract (web summary)

The existence of chemical medium-range order (CMRO) in high- and medium- entropy alloys remains conjectural. Here the authors show evidences of CMRO by electron diffraction spots of lattice periodicity, observable entities, occupancy of preferential species, and stable sizes upon deformation.

## Introduction

High-/medium-entropy alloys (H/MEAs), since their advent^1,2^, have been arousing increasing interest^3–11^. For this new field, what makes H/MEAs structurally different from the traditional alloys has been the first yet hitherto pending question, full of challenges particularly when putting the focus of attention at the atomic scale. The complex atomic-scale heterogeneities inherent to H/MEA demonstrate great potential to trigger the unique microstructures^3–5^. The subnanoscale chemical short-range order (CSRO) is a representative example^12–27^. Recently, the unequivocal evidence of CSRO has been experimentally exhibited in a few H/MEAs^22–27^.

The H/MEAs of multiple principal elements are the complex concentrated solid solution characterized by the high configurational entropy of an ideal solution^1,3–5^. However, enthalpic interaction among constituent species could be sufficiently large in magnitude locally so as to drive the pronounced and widespread local chemical order (LCO) to emerge^19^. LCO in H/MEAs can develop to varying extents/degrees. For example, CSRO, usually less than 1 nm in size, is the incipient LCO^19,25^. Simply following the common idea for a crystal and/or an order to grow up^28^, the chemical medium-range order (CMRO) would be reasonably conjectural. CMRO, here temporarily defined as 1–5 nm in size, is the next-level order structure beyond CSRO. The biggest difference between CMRO from CSRO is the defining crystal structure of periodicity. Instead, CSRO of <1 nm is only a reflection of the preferred occupation of component element species in a much tiny space without a well-characterized lattice structure. In other words, CMRO is the larger LCO in size than CSRO, but still much smaller than the traditional long-range order. CMRO may be taken, by nature, as a significant mark of “gene” inherent to an ordered structure developed in H/MEAs. In view of the structural size and crystal periodicity, CMRO is expected as an indispensable intermediate link between the initial CSRO and final stable long-range phase, usually in the form of an ordered intermetallic compound, i.e., ${{{{{{\rm{L}}}}}}1}_{2}$^9,25^ and ${{{{{\rm{B}}}}}}2$ nano-precipitates, constituting a clearly defined track to understanding the evolution of LCO. Unfortunately, CMRO remains an entirely greenfield thus far in H/MEAs, let alone to catch sight of it. And by the way, CMRO is also a long unascertained structure in the field of metallic glasses^29–31^, still as a tiny crystal to precipitate in an amorphous solution: relative to CSRO, CMRO is large and has a specific crystal structure.

Here, CMRO is explored in an MEA on a few fundamental issues as to firstly, if CMRO would be generated and secondly, if CMRO is mechanically stable. The convincing identification for CMRO, similar to CSRO^25–27^, demands not only the irrefutable diffraction evidence but also intricate chemical details on a sub-/nano-meter length scale regarding the different preferences of constituent species to occupy lattice planes/sites in the next-neighbor atomic shell(s). In the following, CMRO is identified by employing a full suite of characterization methods, including electron diffraction, dark-field imaging, and atomic-resolution chemical mapping. The model material is selected as a dual-phase Al_9.5_CrCoNi MEA^32–34^, consisting of both the face-centered cubic (fcc)-structured base solution and ordered body-centered-cubic (bcc) B2 intermetallic compound, Supplementary Fig. 1.

## Results

### CMRO by electron diffraction and dark-field imaging

**Fig. 1:**
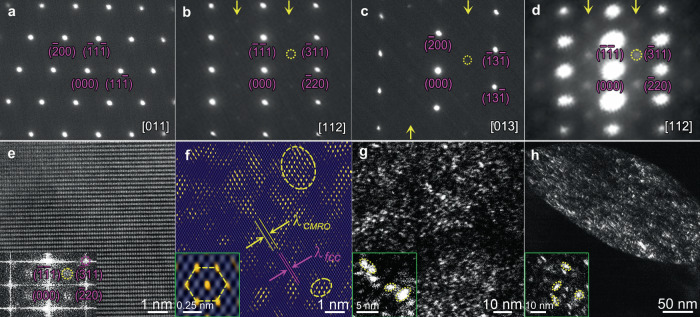
Electron diffraction and dark-field image of CMRO in as-annealed fcc grains. **a**–**c** Selected-area electron diffraction (SAED) patterns under the [$011$], [$112$], and [$013$] z.a. respectively. **d** Nano-beam electron diffraction pattern (NBED). [$112$] z.a. Yellow arrows in **b**–**d**: arrays of extra superlattice reflections. Yellow circle: extra r**e**flection. **e** Lattice image. [$112$] z.a. Inset: FFT pattern. Yellow circle: extra reflection. Pink circle: Braggs spot of fcc phase. **f** IFFT image overlaid fcc lattice image, showing CMRO (circled) and CSRO regions. ${\lambda }_{{CMRO}}$ and ${\lambda }_{{fcc}}$: spacing of {311} plane for CMRO and fcc base. Inset: Close-up view of CSRO (<1 nm). **g,** **h** DF images taken by extra reflections in **d** and **c**, showing superlattice entities. Insets in **g** and **h**: close-up view of CMRO regions.

The selected-area electron diffraction (SAED) pattern under the varying zone axis (z.a.) acts as one of the most fundamental evidence of CMRO. To begin with, under the [$011$] z.a., there is no sign of diffraction information from any superlattice structure, Fig. 1a, except for a set of sharp diffraction spots from the fcc base. Under the [$112$] z.a., Fig. 1b, the extra superlattice reflections appear in the form of many weak spots in intensity, locating at the $\tfrac{1}{2}$[$\bar{3}11$] positions of fcc spots, as indicated by arrows (one is circled in yellow). This indicates doubtlessly the presence of coherent superlattice entities of lattice periodicity in fcc base^35,36^. Now that these reflections emerge at the $\tfrac{1}{2}$[$\bar{3}11$] positions, the [$013$] z.a. would be expected equally appropriate to catch sight of them also. Sure enough, these extra reflections turn up, Fig. 1c, even much weak in intensity, still with an identifiable contrast. The [$013$] z.a. thus serves successfully as verification and complement to the [$112$] z.a. For an improved signal-to-noise ratio^25^, the nano-beam electron diffraction (NBED) pattern is also shown, Fig. 1d, still under the [$112$] z.a. Similar to Fig. 1b, here the extra reflections (arrows) are easily discernible in the form of disks, all still lining at the $\tfrac{1}{2}$[$\bar{3}11$] positions.

Big differences are noted of these extra reflections from those by CSRO previously reported^25–27^. Firstly, the signature diffraction feature by CSRO is the completely diffuse scattering in terms of SAED patterns, usually in the form of disks of large diameter^25–27^. Instead, diffuse scattering vanishes entirely here, see Fig. 1b and c, shifting to the extra spots of concentrated diffraction intensity. Secondly, these reflections, i.e., spots, are much weaker in intensity than sharp spots from fcc spots, still see Fig. 1b and c. Finally, these disk-shaped reflections are rather strong in intensity, even a little bit sharp, in the NBED pattern (Fig. 1d), by comparison to the much weak disks by CSRO under the same imaging conditions^25^. It is, thus, speculated that based on the definitive relationship/correspondence between real and reciprocal space^35^, these weak extra spots are ascribed to the superlattice CMRO entities: they are beyond CSRO in size, but still much tiny in size. Yet, significant similarity is noted that extra reflections by CMRO locate also at the same $\tfrac{1}{2}$[$\bar{3}11$] positions as those by CSRO in other H/MEAs previously reported^25–27^.

Now it is turn to catch sight of these CMRO entities. A high-resolution lattice image-based method was applied via the transition from the fast Fourier transform (FFT) pattern to inverse FFT (i.e., IFFT) imaging. Figure 1e is the lattice image with the [$112$] z.a. Inset is the FFT pattern. The superlattice reflections appear at similar $\tfrac{1}{2}$[$\bar{3}11$] positions; one is circled in yellow as an example. Figure 1f is the corresponding IFFT image using extra reflections overlaying the fcc lattice image by {$311$} spots. Interestingly, the vast majority of superlattice entities cover regions of >1 nm in size, two are circled in yellow. The minor amounts of CSRO of <1 nm in size exist, see inset of a CSRO (labeled by the hexagon). Accordingly, these are definitely the CMRO entities. Further, the {$311$} planes to characterize the lattice periodicity in CMRO (see a pair of yellow parallel lines) have an inter-planar spacing (${{{{{{\rm{\lambda }}}}}}}_{{CMRO}}$) twice that of fcc lattice (${{{{{{\rm{\lambda }}}}}}}_{{fcc}}$, pink parallel lines), Fig. 1f. This is exactly the reason why the extra reflections appear at the $\tfrac{1}{2}$[$\bar{3}11$] positions in the diffraction pattern, see Fig. 1b-d. CMRO is further seen in the energy-filtered TEM dark-field (DF) image, Fig. 1g, and traditional DF image, Fig. 1h, both taken using extra spots in Fig. 1d and c, respectively. Two insets are close-up views for the coherent CMRO entities.

**Fig. 2:**
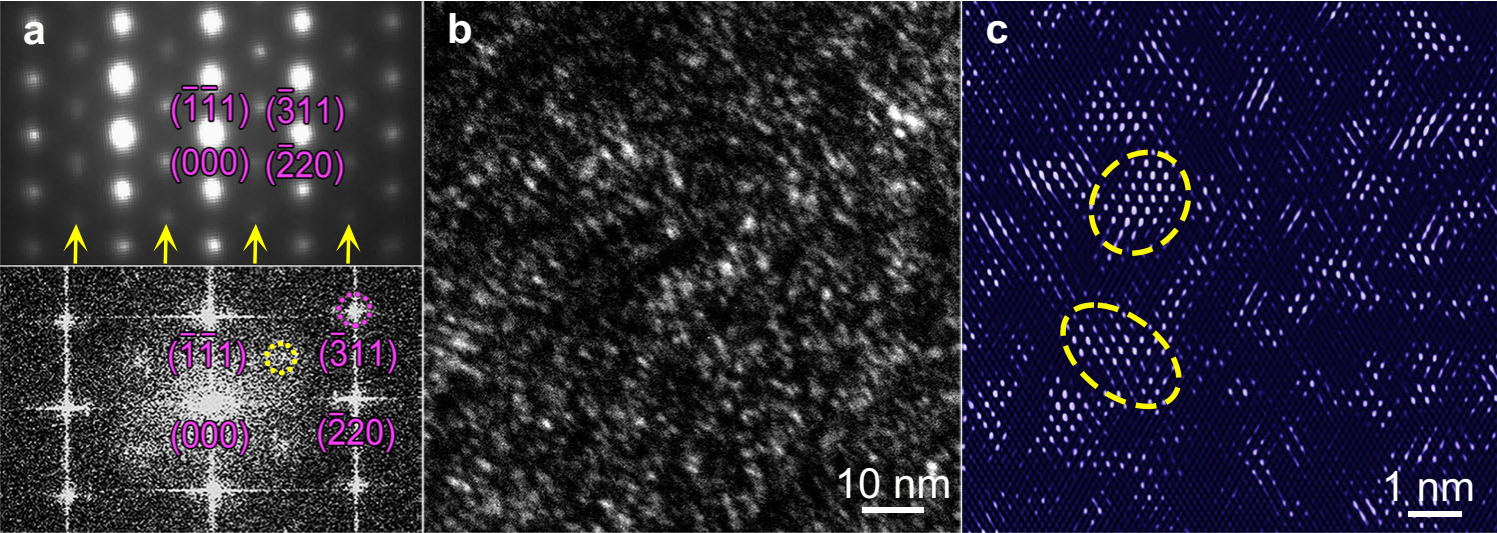
CMRO after tensile deformation. **a** NBED (upper panel) and FFT (lower) patterns. [$112$] z.a. Yellow arrows/circle: array of /one superlattice reflection(s). **b** DF image showing bright entities of CMRO regions. **c** IFFT image. Circles: CMRO regions.

The CMRO entities are further characterized after tensile deformation, Fig. 2. Under the [$112$] z.a., both the NBED (upper panel, Fig. 2a) and FFT (lower panel) patterns show again the extra superlattice scattering at the $\tfrac{1}{2}$[$\bar{3}11$] positions, consistent with those before tensile testing (Fig. 1). Similarly, both DF (Fig. 2b) and IFFT images (Fig. 2c) show the CMRO entities.

### Size distribution and areal density of CMRO

**Fig. 3:**
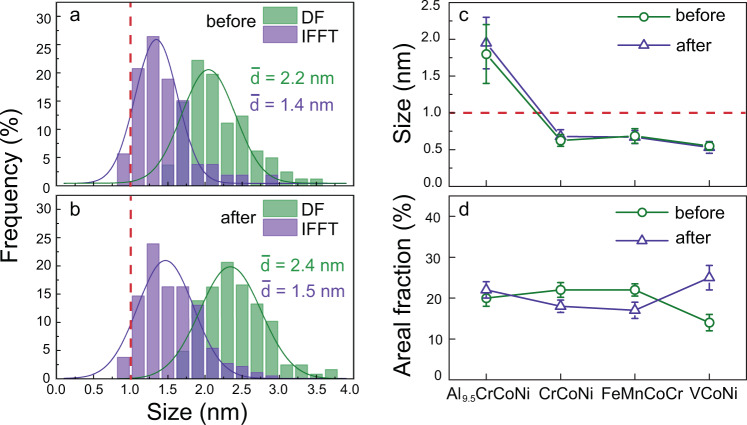
Average size and areal fraction of CMRO. **a**, **b** Size distribution before and after tensile deformation. **c**, **d** Comparison of average size ($\bar{d}$) and areal fraction (${F}_{{areal}}$). The error bars in **c** and **d** donate standard deviation.

Figure 3a and b is the statistic size distribution of CMRO based on both IFFT and DF images before and after tensile deformation. The vast majority is the CMRO entities of sizes >1 nm, with an average size ($\bar{d}$) of 1.8 nm (the average of two averages) and 2.0 nm, respectively. Interestingly, both CMRO (>1 nm) and CSRO are co-existent. Clearly, $\bar{d}$ of CMRO is larger in the present Al_9.5_CrCoNi than that of CSRO in other three H/MEAs^25–27^, Fig. 3c. The increase in $\bar{d}$ is noted after tensile deformation probably due to the mechanically-driven growth. This needs further study to reveal the growth mechanism of these CMRO entities. The areal fraction (${{{{{{\rm{F}}}}}}}_{{{{{{{\mathrm{areal}}}}}}}}$) of CMRO entities is ~20% before tensile deformation, Fig. 3d, which is almost unchangeable after tensile deformation. ${{{{{{\rm{F}}}}}}}_{{{{{{{\mathrm{areal}}}}}}}}$ is comparable to those in both FeMnCoCr^26^ and CrCoNi^27^, except for VCoNi showing a little bit increase^25^.

### Species occupation in CMRO

**Fig. 4:**
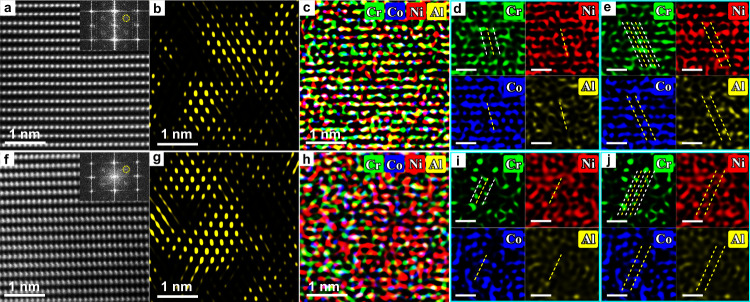
Chemical species occupation in CMRO by EDS mapping. **a** HAADF image under [$112$] z.a. before tensile deformation. Inset: FFT pattern. **b** Corresponding IFFT image showing CMRO regions. **c** EDS map. **d,** **e** Close-up views of Cr, Ni, Co, and Al map, respectively, in an individual CSRO in **d** and CMRO region in **e**. All dashed white/yellow lines: Cr-enriched and Cr-depleted {$\bar{3}11$} planes. **f**–**j** Corresponding results after tensile deformation. Scale bars in (**d**, **e**, **i**, and **j**): 0.5 nm.

The chemical information of CMRO is investigated by probing into specific arrangements of four chemical species, i.e., Cr, Co, Ni, and Al. Based on HAADF imaging under the [$112$] z.a. before tensile deformation, Fig. 4a, the IFFT image was available, showing CMRO entities, much close mutually, with sizes larger than 1 nm, Fig. 4b. Along the horizontal direction in Fig. 4a, the spatial distribution of each individual element was monitored from column to column to obtain the corresponding EDS mapping, Fig. 4c. Refer to Supplementary Fig. 2 for total 15 independent line scan profiles for Cr, Co, Ni, and Al, respectively. One atomic column along the thickness direction of TEM foil produces a colored spot, Fig. 4c. The make-up of specific species in the column determines the intensity of each spot, relating closely to the content of a probed element. Interestingly, Cr atoms demonstrate the same occupancy in both CSRO and CMRO, see green spots in two Cr-maps of upper-left panels, respectively, Fig. 4d and e. Specifically, there are two Cr-enriched ($\bar{3}11$) planes (dashed white lines, across green spots) which sandwich one Cr-depleted ($\bar{3}11$) plane under the yellow line. The latter shows the intense red/blue/yellow spots (see other three panels, respectively, for Ni, Co, and Al), with faint or even vanishing Cr atoms in green. In other words, both CSRO and CMRO form an alternating preferential chemical order that the Cr-enriched ($\bar{3}11$) planes alternate with Cr-depleted (i.e., Co/Ni/Al-enriched) ones. This makes a period of ($\bar{3}11$) inter-planar spacing in CMRO which doubles that of fcc lattice. This offers an explanation again that the $\tfrac{1}{2}$[$\bar{3}11$] locations are where superlattice reflections should appear in all SAED and NBED patterns. The perfectly consistent results after tensile deformation are shown in Fig. 4f-j. Here once again, CMRO (Fig. 4j) shows the same occupation of chemical species as that in CSRO (Fig. 4i). This chemical occupancy extends from a few ($\bar{3}11$) planes at a distance <1 nm in CSRO (Fig. 4d and i) to more planes >1 nm in CMRO (Fig. 4e and j).

## Discussion

CMRO entities are verified, which are co-exist with CSRO ones. The conventional SAED pattern by CMRO is different markedly from that by CSRO. The iconic diffraction for CSRO is the diffuse scattering in the form of large-sized disks^25–27^. Instead, the counterpart by CMRO becomes spots, Fig. 1b and c. This radical change to occur in reciprocal space is ascribed from an increase in sizes of CMRO entities as compared to that of CSRO, based on the definitive relationship/correspondence between real and reciprocal space^35^. In other words, CMRO entities are large enough in size to allow the presence of sharp diffraction spots to reflect the periodicity of ordering structures^36,37^. At the same time, these extra spots by CMRO entities are extremely weak in diffraction intensity as compared to sharp base spots, Fig. 1b and c. This is due to the much fewer periodic structures to be detected for CMRO entities.

CMRO is chemically ordered. Of special interest is the full accord in elemental occupation between CMRO and CSRO, by comparing Fig. 4d and e (CSRO and CMRO, respectively, before tensile deformation) and Fig. 4i and j (after deformation). Both the CMRO and CSRO also have the same struture motif of atomic configutation^25,27^, see Fig. 1f. This strongly signals that CMRO is growing CSRO, but at a medium range of LCO. The co-existent CMRO and CSRO lend support to this idea, see Figs. 1 and 2. Yet, it is unexpected that CMRO, along with CSRO, differs from the ordered bcc B2 already existing in this dual-phase MEA. Namely, both CMRO and CSRO of the same structure motif are hard to predict the development of order^25^. In view of the structural size, CMRO, instead of CSRO, can be qualified as the nuclei of a precipitate, even subcritical embryos. However, this CMRO differs from the B2 phase already precipitated. In other words, this fcc base would remain still as fcc; but, it is so concentrated such that CMRO develops from CSRO. This is probably a unique feature of the development of an ordered struture in H/MEAs.

Recently, the chemical ordering in CSRO has been detected in VCoNi^25^, CrCoNi^22,27^, and FeMnCoNi^23,26^. The comparison of CMRO with CSRO is therefore in order. First, chemical ordering in CMRO is in full accord with that of CSRO found in the above three H/MEAs^25–27^: the alternating {311} planes with the preference for unlike pairs and avoidance of unlike pairs. Both holds for the present CMRO and all other CSRO investigated so far. Second, the present CMRO/CSRO shows a similar structure motif of elemental species to that in three H/MEAs^25,27^. The reason for this is still an open issue. The in-depth investigation demands energy computations, which is beyond the scope of this experimental study. Third, CMRO emerges. This is expectable due to the ${{{{{\rm{B}}}}}}2$ formation by the negative heat of mixing in this MEA. The annealing temperature is probably a factor to facilitate CMRO. The degree of CSRO/CMRO should be better established under specific processing conditions. Finally, CMRO changes little in size and areal fraction after tensile deformation. This stresses that CMRO is mechanically stable during tensile deformation, similar to CSRO^25–27^.

In summary, even there are reasons to believe that so far, the convincing demonstrations of chemical medium-range ordering have been missing for the high-/medium-entropy alloys. This work fills this hole. The chemical medium-range order is observed to extend from the short-range order. The selected-area electron diffraction, fast Fourier transform, and nano-beam electron diffraction, with both the [$112$] and [$013$] zone axis, show the extra superlattice scattering by chemical medium-range order in an Al_9.5_CrCoNi medium-entropy alloy. The EDS mapping shows two Cr-enriched {$311$} planes sandwich one Cr-depleted plane based on preferred arrangements of four chemical species across neighboring atomic planes. Further, both CSRO and CMRO of the same structure motif are different from the chemical order of the known second phase (here, B2). From a general perspective, it is anticipated that mechanically stable CMRO, along with CSRO, will enhance the mechanical property. CMRO of large sizes will be more effective to reinforce the interaction with dislocations upon straining. CMRO, along with CSRO, offers a new knob to turn, i.e., by providing an opportunity to tune the degree of chemical orders to tailor the macroscopic properties. The present results suggest a potential strategy to tailor the macroscopic properties via heterogeneous CMRO engineering in H/MEAs.

## Methods

### Materials processing

The Al_9.5_CrCoNi MEA was produced by an arc-melting technique. Pure chromium, cobalt, nickel, and aluminium (all >99.9% purity) were melted and cast into an iron mold of 130 mm diameter under argon atmosphere. The ingot was re-melted five times to ensure homogeneity. The chemical composition (at%) was analyzed to be 9.5%Al, 30.5%Cr, 29.8%Co, and 30.2%Ni. Subsequently, the ingot was hot-forged and hot-rolled at 1150 °C to a thick plate of dimensions of 12 × 80 × 800 mm^3^. This plate was then microstructurally homogenization-treated at 1100 °C for 12 h in a vacuum, followed by rapidly quenching in water. Finally, the plate was cold-rolled to a thin sheet of 1.0 mm thick after a thickness reduction of 90%. The recrystallization annealing was carried out at 1000 °C for 30 min.

### Mechanical properties test

Tensile specimens were cut from the annealed sheet with a longitudinal axis along a rolling direction. The gauge cross-section was 4 × 1 mm^2^ and 15 mm in length. The tensile tests were performed at room temperature and strain rate of 5 × 10^−4^ s^−1^ in an MTS 793 machine.

### Microstructure characterization

The microstructure before tensile straining was then observed using the electron backscattered diffraction (EBSD) imaging in a ZEISS Gemini 300 scanning electron microscope with an EBSD detector. CMRO observations were conducted before and after tensile testing (thin foils were cut from the gauge section). All thin foils were mechanically polished to 50 μm thick, which then punched to discs of 3 mm in diameter for perforation using twin-jet electro-polishing. The observations were performed by the high-resolution transmission electron microscope (HR-TEM), along with an aberration-corrected high-angle annular dark-field (HAADF)-scanning transmission electron microscope (STEM) in an FEI Titan Cubed Themis G2 300 operated at 300 kV, equipped with a Super-X energy-dispersive X-ray spectroscopy (EDS) with four windowless silicon-drift detectors. The nano-beam diffraction was performed under the mode of TEM microprobe, with an electron beam spot diameter of 35 nm. The image was obtained by using the Flucam-Viewer with Sensitivity 6. The atomic-resolution EDS mapping was conducted at the count rate ranging from 180 to 500 cps and the dwell time was 5 μs per pixel with a map size of 512 × 512 pixels.

## Supplementary information

Supplementary information

## Data Availability

The data that support the findings of this study are available from the corresponding author upon reasonable request.
