# Supplementary material for: Chemical medium-range order in a medium-entropy alloy
Source: Nat Commun. 2022 Feb 23;13:1021. doi: 10.1038/s41467-022-28687-w (PMC8866532; doi:10.1038/s41467-022-28687-w)
Supplement: Supplementary file 1 — Supplementary information [file 41467_2022_28687_MOESM1_ESM.pdf]

Supplementary Information

for “Chemical medium-range order in a medium-entropy alloy”

Contents

1. Supplementary Figure 1
2. Supplementary Figure 2

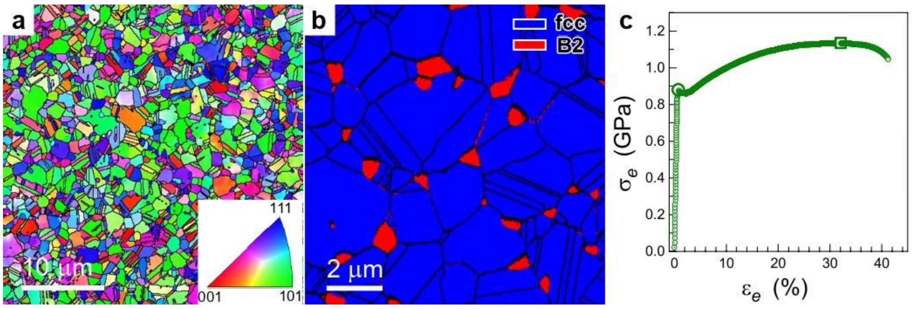

Supplementary Figure 1. As-annealed two-phase microstructure and tensile engineering strain-strain curve in $Al_{9.5}$CrCoNi medium-entropy alloy. a EBSD Inverse Pole Figure (IPF). **b** EBSD Phase image, showing B2 (in red) at grain boundaries of fcc grains (in blue). **c** Tensile engineering stress-strain curve for as-annealed sample at 1273 K for 30 minutes. Circle: yield strength of 870 MPa. Square: ultimate tensile strength (UTS) of 1150 MPa.

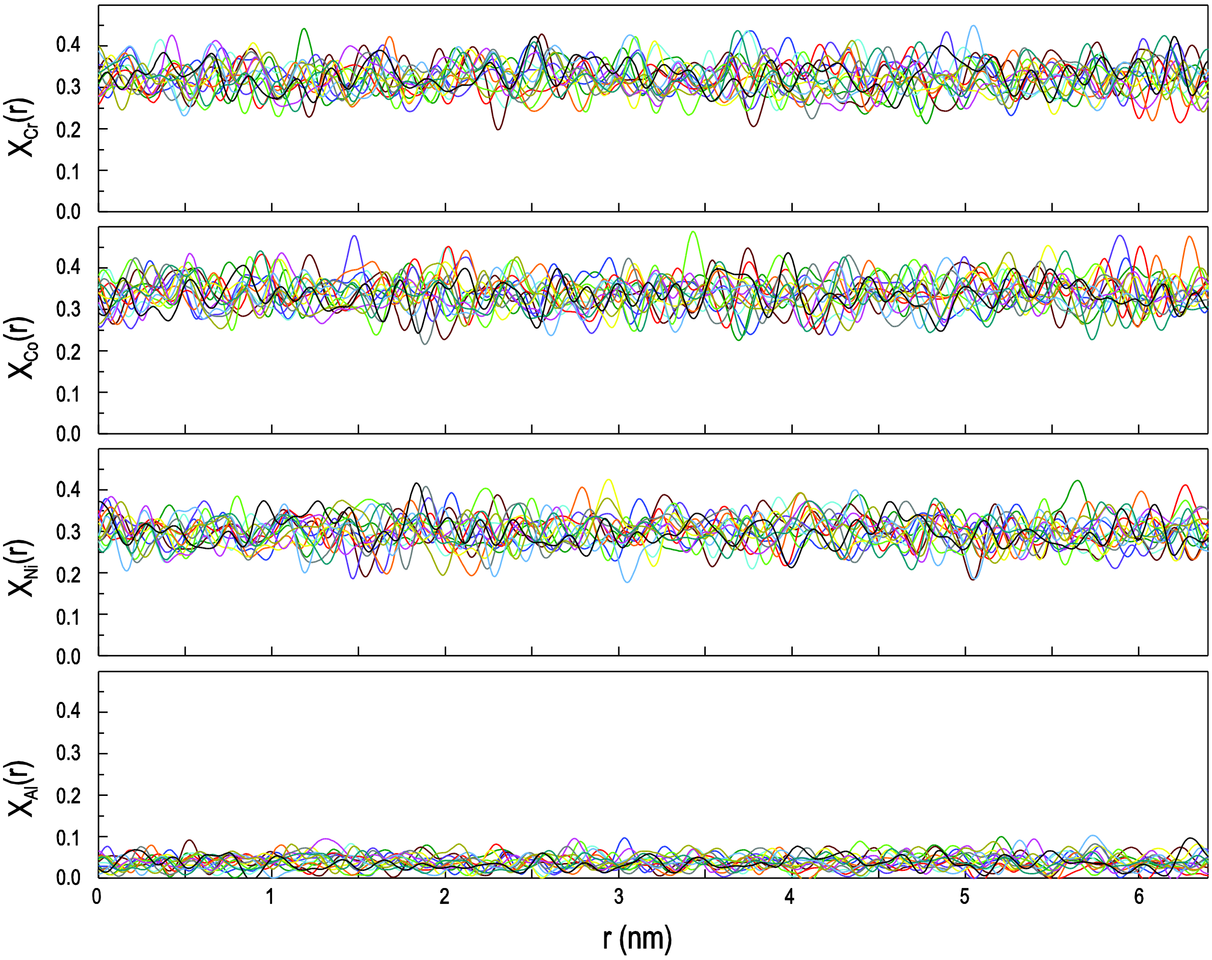

Supplementary Figure 2. Totally 15 independent line scan profiles, respectively, for Cr, Co, Ni, and Al along the horizontal direction in Fig. 4a. Each line profile represents the distribution of an element in a (111) plane, column-by-column, projected along the [112] zone axis. The line with the same color, respectively in 4 figures, corresponds to the same test for 4 elements.
